# Inflammatory myo-fibroblastic tumor of appendix: A rare clinical entity

**DOI:** 10.1016/j.ijscr.2021.106607

**Published:** 2021-11-22

**Authors:** Suman Baral, Om Bahadur Karki, Suman Poudel, Subash Bhattarai, Puskar Poudel

**Affiliations:** a Department of Surgery, Dirghayu Pokhara Hospital Ltd, Pokhara, Nepal; b Department of Surgery, Manipal Teaching Hospital, Pokhara, Nepal; c Department of Pathology, Gandaki Medical College, Pokhara, Nepal; d Department of Medical Gastro-enterology, Manipal Teaching Hospital, Pokhara, Nepal; e Matrisishu Miteri Hospital, Pokhara, Nepal

**Keywords:** Acute appendicitis, Inflammatory myo-fibroblastic tumor

## Abstract

**Introduction:**

Inflammatory myo-fibroblastic tumor of appendix is one of the rarest clinical findings and less has been described in the literatures. So, we aimed to present the clinical case that we encountered at our institute.

**Presentation of a case:**

A 29-year-old lady presented with history of pain at the periumbilical area for one day which shifted to right iliac fossa. Clinical examination revealed tenderness and rebound at right iliac fossa with increased total leucocyte count and ultrasonography abdomen showed swollen appendix. Intraoperatively, a lump around 4 × 3 cm was evident at the tip of appendix with cut section revealing pus along with fecalith. Appendectomy was done with no spillage of the content within the peritoneal cavity. Histopathology revealed inflammatory myo-fibroblastic tumor of appendix. Patient is on regular follow up.

**Discussion:**

Inflammatory myo-fibroblastic tumor of appendix is one of the rarest clinical findings that mimics malignancy. Presentation could be that of acute appendicitis and in most instances, diagnosis is made intraoperatively. Surgical removal is the mainstay of treatment with regular follow up of the patient for chances of recurrences. Histologically, edematous stroma consisting of inflammatory infiltrates composed of lymphocytes, plasma cells, eosinophils and focal formation of lymphoid follicles along with the proliferation of scattered spindle to ovoid cells with proliferating blood vessels with unremarkable over lying epithelium is evident. Myo-fibroblastic origin can be confirmed by immunostaining with smooth muscle specific vimentin and actin.

**Conclusion:**

Inflammatory myo-fibroblastic tumor of appendix can present with features of acute appendicitis and may mimic malignancy. Appendectomy with regular follow up is mandated if such clinical cases are encountered.

## Highlights

• Inflammatory myo-fibroblastic tumor is a pseudo sarcomatous condition
• Appendiceal involvement is pretty rare.
• Clinical presentation may mimic that of acute appendicitis.
• Surgical removal and regular follow up are the main stay of treatment.

## 1 Introduction

Inflammatory myo-fibroblastic tumor (IMT) belongs to a group of pseudo-sarcomatous conditions with various designations including inflammatory pseudotumor, plasma cell granuloma, plasma cell pseudotumor, myo-fibro-histiocytic proliferation etc. [1], [2]. This is a histologically idiosyncratic lesion characterized by myofibroblast proliferation and inflammatory cell infiltration involving most commonly lungs whilst extra pulmonary involvement as gastrointestinal tract is very rare and usually predilects younger age groups [3]. We believe this to be the fifteenth case of IMT involving the appendix [4]. We hereby describe this rare clinical entity in a 29-year old lady who was incidentally diagnosed with IMT while operating for features of acute appendicitis. The work has been reported in line with the SCARE 2020 criteria [5].

## 2 Case presentation

**Fig. 1:**
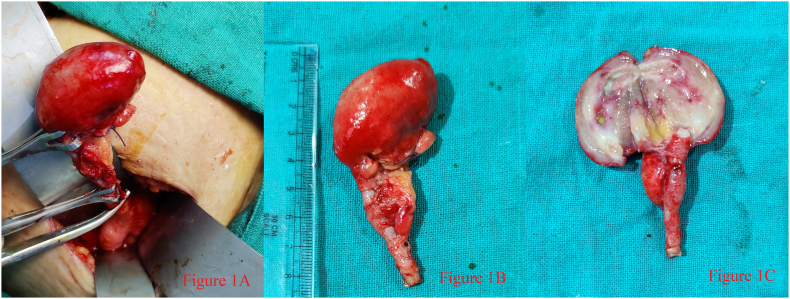
**A** shows the tip of the appendix with the lump at right iliac region along with the base at caecum. **Figure 1B** shows the gross specimen of the appendix including the tip measuring approximately 4 × 3 cm in size. **Figure 1C** shows the cut surface of the lump with grey white mucoid area with yellow colored fecalith within.

A 29-year-old lady presented to the surgical out-patient department with the complaints of pain at the periumbilical region for one day which later shifted to the right iliac region. Pain abdomen was associated with 2–3 episodes of vomiting and anorexia. There was no history of fever, burning micturition, black-colored stool or passage of reddish urine. She gave no history of such illness among the family members, not any history of surgery in the past. She took pain killers for the associated pain. Clinical examination revealed tenderness on palpation in the right iliac fossa with rebound tenderness. Laboratory examination showed total leucocyte counts of 11,300/mm^3^ with neutrophilia (80%) and serum amylase of 98 U/l. Ultrasonography abdomen showed features of acute appendicitis with wall-to-wall diameter of 8 mm along with minimal free fluid in the pelvic and right iliac region. In view of the diagnosis of acute appendicitis, the patient was planned for open appendectomy as laparoscopic facilities were not available. The appendix was positioned in pelvic position with around 50 ml of collection in pelvis, inflamed along with a swelling around 4 × 3 cm involving the tip of the appendix. (Fig. 1A and B) In view of probability of carcinoids or malignancy, inflammatory fibroid polyps, gastrointestinal stromal tumors and lymphoma, the spillage of the appendiceal content was controlled and the lesion was removed enbloc with the meso-appendix. No enlarged mesenteric lymph nodes were evident. The cut section of the tip revealed a grey white mucoid area with 3 ml of pus and a faecalith. (Fig. 1C) Pus culture sensitivity showed growth of *E coli* sensitive to Nitrofurantoin and Cotrimoxazole. The patient was discharged on 2nd post-operative day without complications.

**Fig. 2:**
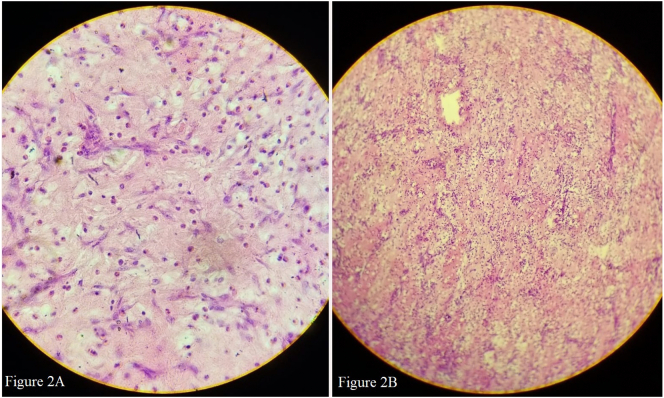
**A**- H&E stained 400X: Section shows plump myofibroblast with eosinophils, lymphocytes and capillary proliferation. **Figure 2B**- H&E stained 100X: Section shows edematous stroma with mixed inflammatory infiltrates.

Histopathological examination of the specimen showed submucosal edematous stroma consisting of inflammatory infiltrates composed of lymphocytes, plasma cells, eosinophils and focal formation of lymphoid follicles. The proliferation of scattered spindle to ovoid cells with plump nucleus and small nucleoli resembling myofibroblasts along with proliferating blood vessels with unremarkable over lying epithelium were evident suggestive of Inflammatory myo-fibroblastic tumor. (Fig. 2A and B) Post-surgical follow up in 6 months showed no any evidences of recurrence. The patient continues to be followed up in the clinic.

## 3 Clinical discussion

Inflammatory myo-fibroblastic tumors (IMT) are one of the rare clinical findings histopathologic ally showing dense inflammatory cells with myofibroblast proliferation. Lungs are the common sites of their involvement, however extrapulmonary involvement are also evident involving almost all systemic components of the body [3], [6], [7], [8]. Gastrointestinal involvement is rare and cases have been published regarding involvement of stomach, appendix, colon, mesenteries, pancreas, liver and omentum [9]. Less than fifteen cases on involvement of appendix have been mentioned in the literatures. Currently, there has been doubt about the originality of the lesion considering it to be neoplastic with intermediate malignant potential in the recent classification by WHO evidenced by chromosomal aberration in 30–40% of cases suggesting clonal origin associated with aggressive clinical behavior [1].

The true incidence of the lesion is unknown as most of the cases go unnoticed as immunostaining is not routinely done or is not available in most instances especially at low resource settings that predisposes to under reporting of the disease. Among 74 abdominal cases of IMTs in children which was reviewed by Bonnet et al. [10], appendiceal origin was found in a single case of 8-year-old boy which was reported by Narasimharao et al. [11] Neutrophilia with leukocytosis was evident in a case reported by Bashir et al. [1] which was also observed in a case by Occonomopoulou et al. [2] as in our case too. Exaggerated or aberrant tissue response to chronic inflammatory process occurring after infection, trauma or surgery are considered the etiopathogenesis whilst published literatures show the agents like *Helicobacter pylori*, *Escherichia coli*, *Epstein Barr Virus*, *Coxiella brunetti*, *Klebsiella pneumoniae* etc. as the infectious agents. Association with Hodgkin disease, peptic ulceration, Behcet disease also has been reported [4].

Vijayraghavan et al. demonstrated the involvement of mid part of appendix with the lesion size of 6 cm along with a fecalith measuring 13 mm as our case demonstrated too [12]. Tip was involved in our case with the similar size of the lump along with presence of fecalith. Bonnet et al. in 1996 published a clinical case of IMT of appendix in a 15-year-old boy who was previously treated for inherited renal tubular acidosis. Clinically, bilateral hydroureteronephrosis with poorly functioning right kidney with retro-vesical soft tissue mass was evident. However, laparotomy revealed the mass arising from appendix which complicated involving the ureters leading to urological signs. Removal of appendix along with the mass sorted the urological issues as well in the next 3 months [10]. Occonomopoulou et al. demonstrated in their case of IMT which involved terminal ileum and ascending colon which mimicked as acute appendicitis [2]. Appendiceal perforation in a patient with IMT of appendix also has been mentioned in the literatures. Kumar et al. found the appendiceal involvement at the tip with the mass of 3 × 3 cm with perforation at the base of the mass along with fecalith at the tip of appendix [13]. Saravanan et al. found the synchronous involvement of the liver along with the appendix when the liver lesion was thought to be hepatocellular carcinoma whilst the diseased appendix was diagnosed and removed by chance. Histopathology of both the specimens revealed IMT [14].

Clinical presentations may include as per the organs involved. Appendiceal involvement may elucidate features of acute appendicitis like pain at right iliac region, anorexia, vomiting though the definitive clinical feature, may not be evident. Laboratory and radiological investigations are not conclusive in most of the instances whilst definitive diagnosis is made on the histopathological examination of the resected specimen [1]. The mainstay of treatment remains the complete surgical excision with regular follow up till a year due to chances of local recurrence. As the lesion corresponds to malignancy anatomically, careful dissection and en block removal is warranted avoiding the spillage of the appendiceal content into the intraabdominal cavity. Microscopically, the lesion consists of myo-fibroblastic spindle cells with inflammatory infiltrations with plasma cells, lymphocytes and eosinophils [2], [3]. Myofibroblastic origin can be confirmed by immunostaining with smooth muscle specific vimentin and actin which shows the positivity [15]. However, due to unavailability of the resources and financial burden to the patient, immunostaining could not be done. The patient is on regular follow up for chances of recurrence. This manuscript aims to create awareness of this type of tumor in the differential diagnosis of appendiceal masses which can avoid overtreatment with hemicolectomies in the view of appendiceal malignancies. Also, this highlights the need of long-term follow-up regarding the tendency for local recurrence and small risk of distant metastasis.

## 4 Conclusion

Inflammatory myo-fibroblastic tumor of appendix could present with features of acute appendicitis and histological assessment is necessary to rule out the possibility of malignancy as the gross anatomical features look alike. Surgical removal along with regular follow up is the mainstay of treatment.

## Provenance and peer review

Not commissioned externally peer reviewed.

## Sources of funding

This case report did not receive any specific grant from funding agencies in the public, commercial, or not-for-profit sectors.

## Ethical approval

Ethical approval was not mandatory for publication of case reports as per the institutional policy.

## Consent

Written informed consent was obtained from the patient for publication of this case report and accompanying images. A copy of the written consent is available for review by the Editor-in-Chief of this journal on request.

## Author contribution

Design and Idea: Suman Baral, Om Bahadur Karki, Subash Bhattarai.

Drafting: Suman Baral, Puskar Poudel, Suman Poudel.

Final Revision: Suman Baral, Om Bahadur Karki, Suman Poudel, Puskar Poudel, Subash Bhattarai.

## Registration of research studies

N/A.

## Guarantor

Suman Baral.

## Declaration of competing interest

Authors declare that there are no any conflicts of interest regarding publication of the manuscript.
